# Postictal Rash: A Rare Case Reporting to Neurology

**DOI:** 10.7759/cureus.69719

**Published:** 2024-09-19

**Authors:** Hamza Jamil, Bridgette King, Rund Radi Haddadin, Muhammad Inam Ul Haq, Maira Tauqeer Pasha, Syed Hashim Ali Inam, Paul Ferguson, Justin Nolte

**Affiliations:** 1 Neurology, Army Medical College, Rawalpindi, PAK; 2 Neurology, Marshall University Joan C. Edwards School of Medicine, Huntington, USA; 3 Neurology, Combined Military Hospital, Rawalpindi, PAK

**Keywords:** epileptic seizures, generalized rash, postictal complications, postictal petechiae, seizures

## Abstract

Generalized epileptic seizures are usually followed by a postictal phase that is often characterized by drowsiness, lethargy, weakness, and confusion. In rare cases, it can present with cutaneous manifestations. Here, we present the case of a 45-year-old male who experienced a seizure and subsequently developed a pinpoint rash with non-blanchable petechiae on various parts of his body. The rash appeared during transport to the emergency department and was resolved after seven days once the seizures were controlled. Initial imaging and basic labs were unremarkable other than a slight increase in postictal markers. No other cause behind this new rash was identified. Our case emphasizes the importance of postictal skin manifestations to aid in diagnosing seizures and avoid unnecessary investigations. The possible pathophysiology behind these generalized non-blanchable petechiae has been attributed to hemodynamic changes and neurogenic inflammation triggered in response to the seizures. Further research into the underlying mechanism and prompt recognition of these symptoms can improve the management of epilepsy care.

## Introduction

The postictal phase is the time following an epileptic seizure [1]. This phase is commonly characterized by focal weakness, as seen in Todd’s paralysis, drowsiness, and confusion [1]. Several cutaneous manifestations have been observed following a seizure such as lacerations, petechiae, and ecchymosis [1]. This type of rash has been described as postictal thoracocervicofacial purpura in some studies [2]. Although postictal rash has been documented by neurologists in a few case reports, it is not commonly known in a hospital setting. Hence, knowledge of postictal cutaneous signs is important for emergency physicians, medicine, and dermatologists so that they can be recognized and treated accordingly without unnecessary investigations [2,3]. The exact mechanism of these cutaneous findings is not fully understood but some suggest that it may be due to high blood pressure or venous congestion during the tonic-clonic phase of a seizure [1].

Here, we present a case of postictal rash after an episode of seizure that resolved once the seizures were controlled.

## Case presentation

A 45-year-old male with a past medical history of subarachnoid hemorrhage secondary to a ruptured posterior communicating artery aneurysm presented to the emergency department (ED) for seizure-like activity. He reported that he woke up in the morning and went to the kitchen and the next thing he remembered was waking up in the ambulance. This event occurred around nine years after the prior subarachnoid hemorrhage episode. According to his wife, the patient had a “staring episode” in the kitchen during which she felt that he was “spaced out,” which was followed by a seizure-like episode in which he collapsed to the floor and fell onto his back. The patient developed seizure-like activity and exhibited jerking of his arms and legs for a few minutes with subsequent postictal confusion and amnesia of the entire event. There was associated tongue biting but no urinary or fecal incontinence. The patient did have a history of subarachnoid hemorrhage nine years ago, for which he was admitted. The etiology of the hemorrhage was secondary to a ruptured aneurysm. He reported having episodes of headaches after undergoing the coiling procedure in 2015 for his aneurysm. He stated that he was using medical marijuana for headaches which had helped him. The patient quit smoking 20 years ago and did not drink alcohol. He had no pertinent family history. This was a first-time seizure event.

When the patient was being transferred from his home to the hospital via ambulance, his wife noticed a pinpoint rash on his face, neck, upper chest, back, and arms which she had never seen before. The patient also denied having a rash before this current episode. The rash was less than 3 mm in size, not palpable, and non-blanchable. The patient denied any recent trauma, recent new drug use, or blood pressure fluctuations. Lab testing did not reveal any qualitative or quantitative platelet abnormalities. On taking extensive history, performing extensive tests, and consulting dermatology, a diagnosis of postictal petechiae was made.

**Figure 1:**
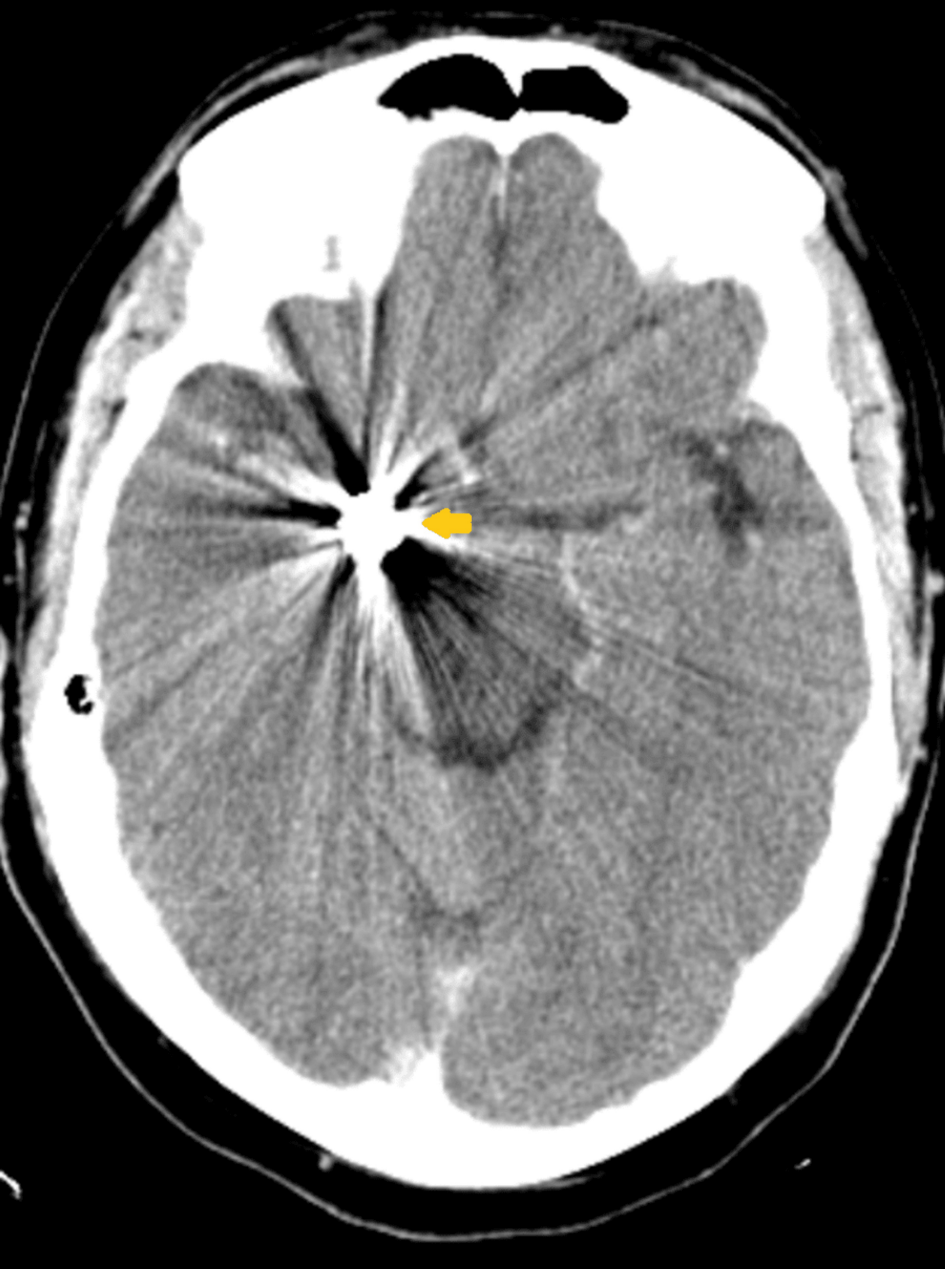
CT of the head showing status post-coil embolization of a right posterior communicating artery aneurysm, with extensive associated metallic streak artifact (yellow arrow), obscuring adjacent structures. No acute territorial infarct. R: right; L: left

On arrival at the ED, the patient developed another seizure with a similar semiology to the prior one, for which he was loaded with levetiracetam and continued on 750 mg IV twice daily levetiracetam. CT of the head showed right posterior communicating artery coiling with right parietal encephalomalacia (Figure 1).

The patient’s lactic acid was 10.5 mmol/L, creatinine kinase was >800 U/L, creatine was 1.8 mg/dL, and white blood count was 27.0 × 10^9^/L initially that improved to 14.5 × 10^9^/L the following day, platelet count was 150 × 10^9^/L, sodium was 134 mmol/L, potassium was 4.9 mmol/L, bicarbonate was 17 mmol/L, prothrombin time was 11.49 seconds, international normalized ratio (INR) was 1.05, and calcium was 9.1 mg/dL. Testing for Lyme’s disease, mono-spot test, Gram stain for meningococcus, serology for Epstein-Barr virus, cytomegalovirus, hepatitis A, hepatitis B, and hepatitis C were negative. Relevant laboratory findings are shown in Table 1.

**Table 1:** Basic laboratory testing results on admission.

Test	Result	Reference value
Creatinine kinase	>800 U/L	30–200 U/L
Creatine	1.8 mg/dL	0.6–1.2 mg/dL
White blood cell count	27 × 10^9^/L	4.0–11.0 × 10^9^/L
Platelet count	150 × 10^9^/L	150–450 × 10^9^/L
Sodium	134 mmol/L	135–145 mmol/L
Potassium	4.9 mmol/L	3.5–5.0 mmol/L
Bicarbonate	17 mmol/L	22–29 mmol/L
Calcium	9.1 mg/dL	8.5–10.2 mg/dL
Red blood cell count	4.24 × 10^12^/L	Men: 4.7–6.1 × 10^12^/L; Women: 4.2–5.4 × 10^12^/L
Hemoglobin	14.4 g/dL	Men: 13.8–17.2 g/dL; Women: 12.1–15.1 g/dL
Hematocrit	41.90%	Men: 40.7–50.3%; Women: 36.1–44.3%
Lactic acid	10.5 mmol/L	0.5–2.2 mmol/L
Aspartate aminotransferase	60 U/L	10–40 U/L
Alanine aminotransferase	65 U/L	7–56 U/L

**Figure 2:**
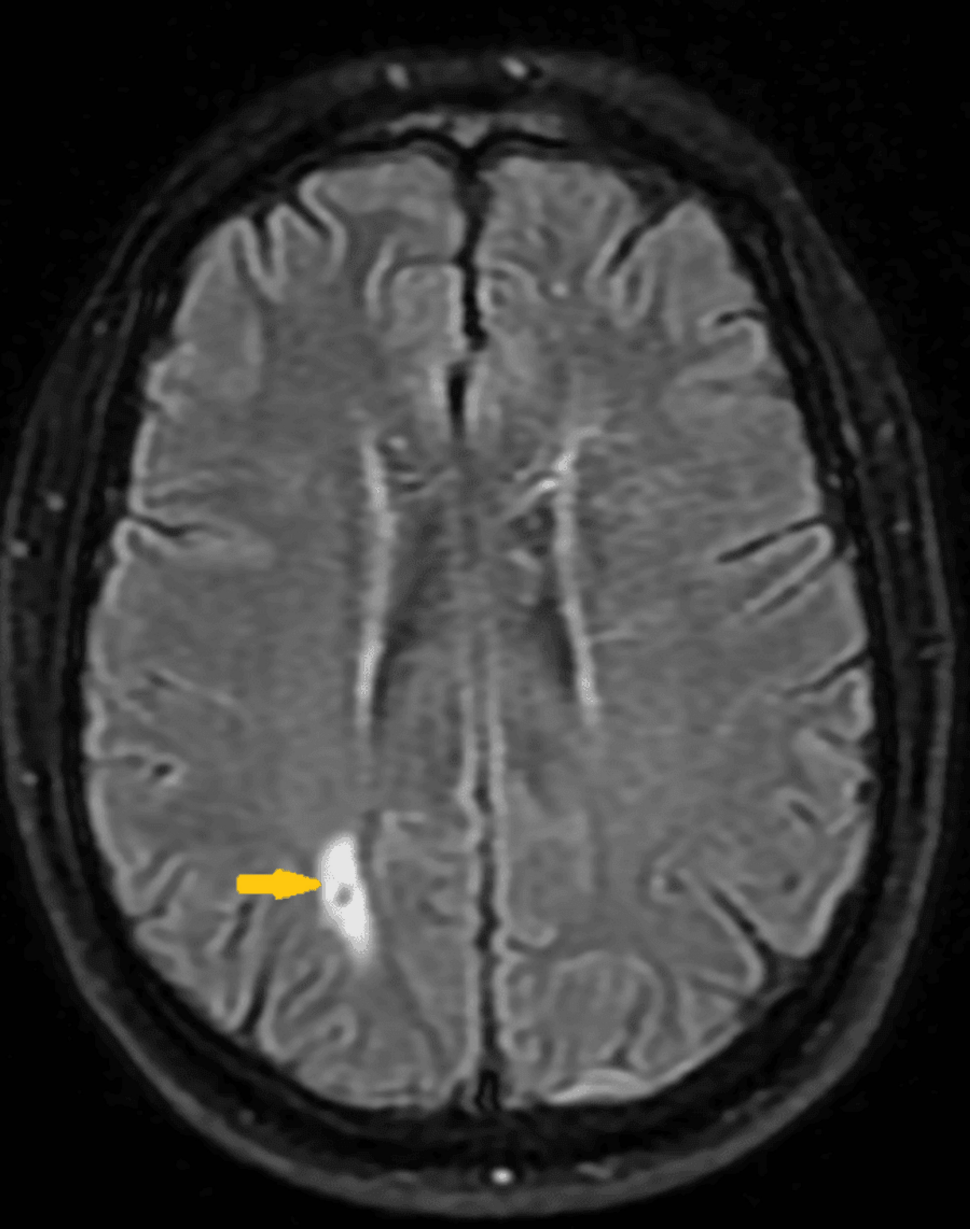
MRI of the brain (T2 FLAIR) showing an area of encephalomalacia with associated susceptibility artifact within the right parietal lobe (yellow arrow). There is no acute infarct. There are no areas of suspicious contrast enhancement. There is no mass effect or midline shift. R: right; L: left; FLAIR: fluid-attenuated inversion recovery

MRI of the brain showed an area of encephalomalacia with associated susceptibility artifact within the right parietal lobe, but no acute changes were noted (Figure 2).

The patient was advised to use levetiracetam per oral 750 mg twice daily, avoid driving for six months, avoid medicines that lower the seizure threshold, avoid sleep deprivation, avoid unsupervised swimming, avoid climbing ladders, and avoid operating heavy machinery. The patient had an uncomplicated hospitalization with overnight observation and remained seizure-free on a 750 mg twice-daily dose of levetiracetam and was discharged home on this dose. He reported the resolution of his rash over a week.

## Discussion

The development of a postictal rash, while uncommon, emphasizes the importance of recognizing the relationship between neurological events and subsequent cutaneous symptoms among emergency physicians, internists, and dermatologists so that they can be correctly diagnosed and managed accordingly without extraneous testing and utilization of healthcare resources.

The pathophysiology involved in the development of a postictal rash is not completely understood. Several hypotheses have been proposed to explain the interaction between convulsive seizure-like activity and dermatological symptoms. One current hypothesis describes the physiological changes that occur during the tonic-clonic phase of a seizure, including changes in blood pressure and venous congestion, which could contribute to these postictal cutaneous manifestations [4]. Seizure activity can create increased intra-abdominal and intrathoracic pressures due to the contraction of chest and abdominal muscles [4]. This response can increase blood pressure, leading to capillary injury and the development of small hemorrhages under the skin [4]. It is hypothesized that these hemodynamic alterations could lead to the development of skin manifestations such as petechiae and ecchymosis that can be observed postictally [4].

Additionally, the role of neurogenic inflammation has also been hypothesized as the trigger for the development of dermatological diseases such as psoriasis, atopic dermatitis, or eczema [5]. Neurogenic inflammation refers to the release of inflammatory mediators from peripheral nerve endings following neuronal stimulation or injury [6]. In the context of seizures, the release of vasoactive neuropeptides such as substance P and calcitonin gene-related peptides from sensory nerves may trigger local inflammatory responses within the skin. This could lead to the release of histamine from mast cells causing dilation and an increase in capillary permeability, leading to erythema and edema [6-8]. This theory seems to have limitations, especially in our patient considering the limited extent of the petechiae rather than the expected generalized appearance as per this hypothesis. This is one of the limitations of our study. In addition, interleukin 6 (IL-6), a platelet-modifying inflammatory cytokine that binds to gp130 on platelets, is released following seizure activity. The release of this inflammatory cytokine could potentially lead to the development of vasculitis and thrombus formation postictally [9-12]. The generalized appearance of vasculitis and thrombus formation usually causes a generalized rash that would be different from the presentation of our patient. This would add to the limitation of our study and open a window for further research regarding the presenting pattern and localization of the postictal rash.

Finally, individual predisposing factors, such as genetic susceptibility, vascular abnormalities, or simultaneous medical conditions, may influence the tendency for postictal rash manifestations. In the case described, the patient’s history of a ruptured posterior communicating artery aneurysm and subarachnoid hemorrhage caused increased levels of IL-6 making him predisposed to an inflammatory response.

## Conclusions

The presented case highlights the importance of recognizing and understanding postictal cutaneous manifestations in the context of seizure disorders. While dermatological symptoms may not be frequent occurrences, their presence can offer insights into underlying pathophysiological processes and prompt multidisciplinary collaboration for comprehensive patient care. Through thorough evaluation, targeted interventions, and ongoing monitoring, healthcare professionals can improve the quality of life for individuals affected by epilepsy and associated cutaneous manifestations. Future research will be essential in further understanding the mechanisms underlying postictal rash and enhancing therapeutic strategies for affected patients.
